# Two decades since the fetal insulin hypothesis: what have we learned from genetics?

**DOI:** 10.1007/s00125-021-05386-7

**Published:** 2021-02-11

**Authors:** Alice E. Hughes, Andrew T. Hattersley, Sarah E. Flanagan, Rachel M. Freathy

**Affiliations:** Institute of Biomedical and Clinical Science, University of Exeter Medical School, Exeter, UK

**Keywords:** Birthweight, Fetal growth restriction, Fetal insulin, Genome-wide association studies, Mendelian randomisation, Neonatal diabetes, Pregnancy, Review, Type 2 diabetes

## Abstract

In 1998 the fetal insulin hypothesis proposed that lower birthweight and adult-onset type 2 diabetes are two phenotypes of the same genotype. Since then, advances in research investigating the role of genetics affecting insulin secretion and action have furthered knowledge of fetal insulin-mediated growth and the biology of type 2 diabetes. In this review, we discuss the historical research context from which the fetal insulin hypothesis originated and consider the position of the hypothesis in light of recent evidence. In summary, there is now ample evidence to support the idea that variants of certain genes which result in impaired pancreatic beta cell function and reduced insulin secretion contribute to both lower birthweight and higher type 2 diabetes risk in later life when inherited by the fetus. There is also evidence to support genetic links between type 2 diabetes secondary to reduced insulin action and lower birthweight but this applies only to loci implicated in body fat distribution and not those influencing insulin resistance via obesity or lipid metabolism by the liver. Finally, we also consider how advances in genetics are being used to explore alternative hypotheses, namely the role of the maternal intrauterine environment, in the relationship between lower birthweight and adult cardiometabolic disease.

**Supplementary Information:**

The online version of this article (10.1007/s00125-021-05386-7) contains a slideset of the figures for download, which is available to authorised users.

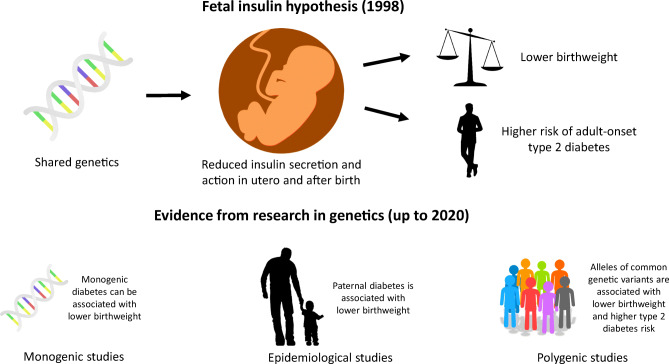

## Introduction

Lower birthweight is associated with a higher risk of adult cardiometabolic disease, including type 2 diabetes [1]. This relationship was first observed in a study from 1991 linking birthweight records to results of glucose tolerance tests performed in adult men [2], and multiple epidemiological studies have since confirmed this association [3]. The ‘thrifty phenotype’ hypothesis was put forward as an explanation in 1992, suggesting that maternal malnutrition led to poor fetal growth, with adaptation to a nutritionally depleted intrauterine environment resulting in abnormal pancreatic beta cell function and reduced capacity to secrete insulin extending into adult life [4]. The thrifty phenotype hypothesis has since expanded to include preconceptual, periconceptual and other intrauterine exposures and postnatal outcomes, and is now known as the Developmental Origins of Health and Disease (DOHaD) hypothesis [5].

**Fig. 1:**
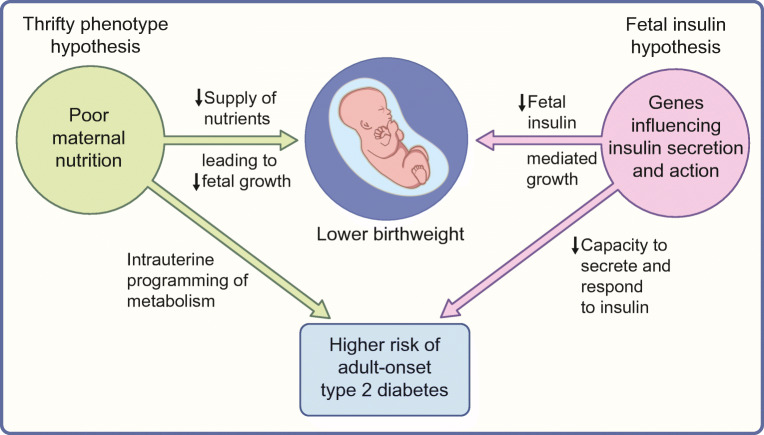
Principles of the fetal insulin hypothesis compared with the thrifty phenotype hypothesis. This figure is available as part of a downloadable slideset

An alternative explanation (the fetal insulin hypothesis) was put forward in 1998, proposing that lower birthweight and adult-onset type 2 diabetes are two phenotypes of the same genotype (Fig. 1) [6, 7]. Jørgen Pedersen identified fetal insulin as a key intrauterine growth factor in 1952 [8] and this, together with the observation that monogenic diseases affecting insulin secretion and action were accompanied by lower birthweight, formed the premise of the fetal insulin hypothesis. It proposed that insulin secretion and resistance, genetically determined and present from conception, also affect intrauterine growth and explain the relationship between lower birthweight and adult-onset type 2 diabetes observed in epidemiological studies [1–3].

In the two decades since the fetal insulin hypothesis was founded, advances in research encompassing the genetics of type 2 diabetes and birthweight have made it possible to test the hypothesis and answer important questions about the relationship between fetal growth and development of type 2 diabetes in later life. In this review, we evaluate the evidence for and against the fetal insulin hypothesis, considering recent evidence from genetic and epidemiological studies. We also consider how genetics could be utilised to explore the complex relationships between the intrauterine environment, fetal genotype and adult-onset type 2 diabetes. The scope of the review does not encompass evaluation of the position of the DOHaD hypothesis in relation to type 2 diabetes risk, as this has been considered in detail in another recent review [9].

## The fetal insulin hypothesis from the perspective of monogenic research

### The role of fetal genotype in determining insulin-mediated growth in utero: studies in families affected by *GCK*-MODY

A study of birthweights from pregnancies affected by MODY due to a heterozygous mutation in the glucokinase gene (*GCK*) [6] provided important insights into how the fetal genotype determines insulin-mediated growth in utero. These mutations result in reduced sensing of glucose by the pancreatic beta cell, so individuals with *GCK*-MODY regulate glucose at a higher set-point (fasting plasma glucose 5.5–8 mmol/l [10]) and have stable, mild hyperglycaemia throughout life [11]. An analysis of birthweights in 23 families with *GCK*-MODY found that where the mother had *GCK*-MODY and her fetus did not, birthweight was approximately 600 g higher than average due to higher fetal insulin secretion in response to maternal hyperglycaemia. However, when the fetus had inherited the *GCK* mutation from their mother, birthweight was no different from average because in such pregnancies glucose is sensed by both mother and fetus at the same level and a normal amount of insulin is secreted. In contrast, where the mother did not have *GCK*-MODY and the fetus had inherited a mutation in *GCK* from the father, birthweight was reduced by approximately 500 g (Table 1). In this case, maternal glucose crossing the placenta is sensed at a higher threshold by the fetus, resulting in less insulin secretion. This work contributed important knowledge to the relationship between maternal blood glucose levels and fetal genotype in regulating intrauterine growth, prompting the proposal of the fetal insulin hypothesis [7].

**Table 1:** Birthweight in monogenic diseases associated with reduced insulin secretion and action

Gene	Disease	Effect on birthweight at term gestation	In support of fetal insulin hypothesis?	References
Reduced insulin secretion				
*GCK*	MODY	↓~500 g	✓	[6]
*HNF1A*	MODY	↔Normal	**✕**	[29]
*HNF4A*	MODY	↑~800 g	**✕**	[29]
*HNF1B*	MODY	↓~800 g	✓	[77]
*ABCC8*, *KCNJ11*	Neonatal diabetes	↓~800 g	✓	[13–16]
Absent insulin secretion				
*INS*	Neonatal diabetes	↓~1500 g	✓	[17]
*CNOT1*, *GATA4*, *GATA6*, *PDX1*, *PTF1A*	Pancreatic agenesis	↓~1500 g	✓	[18–23]
Insulin resistance				
*INSR*	Congenital insulin resistance	↓~1500 g	✓	[33–35]
*AGPAT2*, *BSCL2*, *CAV1*	Congenital generalised lipodystrophy	↔Normal	**✕**	[37, 78–80]
*LMNA*, *PPARG*, *PLIN1*	Familial partial lipodystrophy	↔Normal	**✕**	[38–42]

Studying the genetics of *GCK*-MODY pregnancies to gain knowledge of birthweight has been clinically important as it has informed obstetric care. Historically, these at-risk pregnancies were monitored with serial ultrasound scans and the fetus was assumed not to have inherited the maternal mutation if there was evidence of fetal overgrowth (abdominal circumference >75th percentile for gestational age). In this case, treatment of maternal hyperglycaemia was trialled, followed by planned delivery at 38 weeks gestation to mitigate the intra- and postpartum risks of having a large-for-gestational-age (LGA) baby. More recently, non-invasive prenatal diagnostic testing of cell-free fetal DNA in maternal blood has become available [12] and has the potential to prevent unnecessary treatment of maternal hyperglycaemia in fetuses who have inherited a *GCK* mutation.

### Single-gene mutations that result in reduced insulin secretion typically reduce birthweight

The discovery that neonatal diabetes is commonly caused by mutations in single genes affecting insulin secretion has lent further support to the fetal insulin hypothesis (Table 1) [6, 13–23]. These cases are rare and represent a severe phenotype but the principle that genetics determines both fetal growth and postnatal insulin secretion is supported by the observation that infants with neonatal diabetes have very low birthweights (median SD score (SDS) for sex and gestational age −1.7 [24]). Furthermore, the severity of fetal growth restriction depends on the amount of fetal insulin secretion, as infants with complete absence of fetal insulin secretion due to loss-of-function mutations in the insulin gene or pancreatic agenesis are half of normal birthweight by term gestation (median SDS for sex and gestational age <−3.0, unpublished data from A. Hughes et al). This is in contrast to other animal species, where absent fetal insulin secretion reduces birthweight to a much lesser extent than in humans [25]. Therefore, human birthweight is a bioassay of inherent insulin secretory capacity, and monogenic disorders of insulin secretion provide unique insights into the genetic link between lower birthweight and diabetes resulting from reduced insulin secretion.

### Birthweights in *HNF4A*-MODY and *HNF1A*-MODY are not consistent with the fetal insulin hypothesis

Not all instances of monogenic diabetes secondary to reduced insulin secretion are associated with lower birthweight. Heterozygous mutations in the genes encoding the transcription factors hepatic nuclear factor-4α and -1α (*HNF4A* and *HNF1A*, respectively) result in reduced insulin secretion [26, 27] and mutation carriers develop diabetes in childhood or early adulthood [28]. The fetal insulin hypothesis would predict that affected individuals have a low birthweight, yet individuals with *HNF1A*-MODY have normal birthweights and inheritance of *HNF4A*-MODY is associated with fetal and neonatal hyperinsulinism and macrosomia (Table 1) [29]. It has been proposed that fetal hyperinsulinism causes accelerated postnatal pancreatic beta cell apoptosis, which subsequently predisposes to early-onset diabetes [30]. However, it has recently been found that higher birthweight is associated with reduced penetrance of *HNF4A*-MODY (unpublished data from J. Locke and K. Patel). Therefore, higher birthweight in *HNF4A*-MODY is likely to represent a greater inherent capacity to secrete insulin, and differential expression of *HNF4A* isoforms in the fetus and in later life [31, 32] may provide an alternative explanation for these contrasting effects of *HNF4A* mutations.

### Monogenic diseases resulting in severe insulin resistance have heterogeneous effects on birthweight

The relationship between birthweight and monogenic diabetes secondary to impaired insulin action is unclear (Table 1). Consistent with the fetal insulin hypothesis, infants with severe congenital insulin resistance secondary to loss-of-function mutations in the insulin receptor gene, *INSR*, have very low birthweights [33–35]. Single-gene mutations resulting in either congenital generalised or familial partial lipodystrophy are characterised by peripheral insulin resistance due to an absence of subcutaneous adipose tissue, and affected individuals typically develop diabetes in adolescence [36]. However, birthweights of infants with congenital generalised lipodystrophy have been reported to be normal [37] and though there are reports of low birthweight in familial partial lipodystrophy [38, 39], this has not been widely reported as a typical clinical feature in the literature [40–42].

## The fetal insulin hypothesis from the perspective of epidemiological research

### Paternal type 2 diabetes is associated with lower offspring birthweight but is not clearly related to heritable insulin resistance

**Fig. 2:**
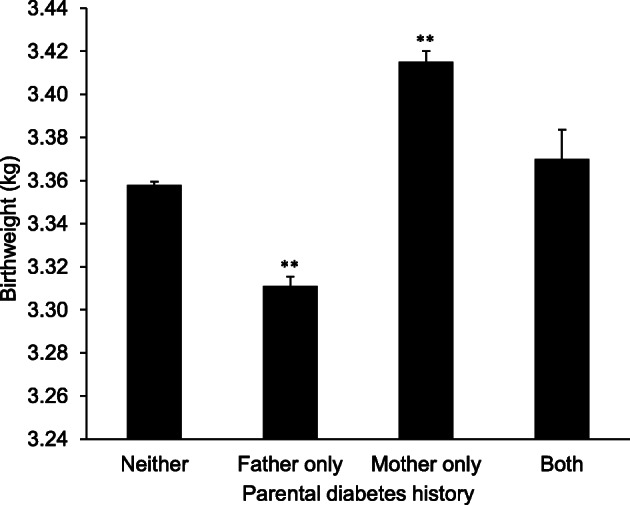
Birthweight according to parental diabetes status in the UK Biobank study [43]. **p<0.001 vs birthweight where neither parent was reported to have diabetes. Figure adapted from Tyrell et al [43] under the terms of the Creative Commons Attribution 3.0 Unported License. This figure is available as part of a downloadable slideset

Observational studies of paternal diabetes status and offspring birthweight have provided evidence for a shared genetic predisposition to lower birthweight and type 2 diabetes [43, 44]. The study of paternal diabetes is important, since maternal diabetes leads to higher birthweight [45] and masks the effect of fetal genes predisposing to diabetes inherited from the father. This was clearly shown by a study of 236,030 participants (UK Biobank study) wherein paternal diabetes was associated with a 45 g lower birthweight compared with birthweights of infants who had no parent with diabetes. In contrast, birthweight in offspring of parents who both had diabetes was not different from birthweight of infants for whom neither parent had diabetes(Fig. 2) [43].

The fetal insulin hypothesis proposed a possible role for heritable insulin resistance, and there has been evidence for a relationship between low birthweight and higher levels of paternal insulin resistance in case–control (*n*=119) [46] and cross-sectional (*n*=2788) [47] studies. However, paternal insulin resistance was not independently associated with offspring birthweight in a birth-cohort study of 986 UK parent–offspring trios [48], and there was a positive correlation between paternal HOMA-IR and umbilical cord insulin levels in 644 fathers and babies [49]. Together, this suggests that in utero there may in fact be a compensatory rise in insulin levels in the face of insulin resistance to maintain fetal growth.

## The fetal insulin hypothesis from the perspective of polygenic research

### Type 2 diabetes risk loci are associated with lower birthweight

The first genome-wide association studies (GWAS) transformed the landscape of research into the genetics of type 2 diabetes [50–52] and allowed us to test the fetal insulin hypothesis. Initially, variants at type 2 diabetes risk loci affecting insulin secretion were tested for their association with birthweight and it was found that fetal risk alleles at the *CDKAL1* and *HHEX-IDE* loci were associated with a lower birthweight [53, 54]. The effect was also important; the reduction in birthweight in a fetus carrying four risk alleles was equivalent to that seen in a fetus whose mother smoked three cigarettes per day in the third trimester of pregnancy.

The first GWAS for birthweight shortly followed [55] and one of the first variants identified was at the known type 2 diabetes risk locus in *ADCY5*, which plays a key role in coupling glucose to insulin secretion from the pancreatic beta cell [56]. Since then, successively larger GWAS of birthweight, with the latest including data on >400,000 individuals, have identified a total of 190 loci associated with birthweight [57–59]. Using a recently developed method [59, 60], the statistical power from these large samples could then be harnessed to estimate the independent maternal and fetal effects at each locus. To date, 11 variants with fetal effects both on birthweight and on type 2 diabetes risk have been identified (Table 2).

**Table 2:** Fetal risk loci associated with birthweight and type 2 diabetes

Birthweight and type 2 diabetes risk locus	Effect of fetal type 2 diabetes risk-raising allele on birthweight (*z* score)	Likely biology underlying type 2 diabetes risk
*IRS1*	−0.02	Higher insulin resistance [81, 82]
*ADCY5*	−0.06	Reduced insulin secretion [81, 82]
*CDKAL1*	−0.05	Reduced insulin secretion [81, 82]
*ANK1*	+0.03	Reduced insulin secretion [81, 82]
*GPSM1*	−0.02	Not known
*HHEX/IDE*	−0.04	Reduced insulin secretion [81, 82]
*PLEKHA1*	−0.02	Not known
*INS-IGF2*	−0.03	Not known
*KCNQ1*	−0.02	Reduced insulin secretion [81, 82]
*CCND2*	−0.01	Reduced insulin secretion [81, 82]
*HMGA2*	−0.04	Reduced insulin secretion [83]

Birthweight SNPs [59] at these loci are in linkage disequilibrium (*R*^2^>0.3) with a primary or secondary signal type 2 diabetes SNP [61]. A 1 SD change in birthweight is equivalent to ~450 g

### There is heterogeneity in the relationship between birthweight and type 2 diabetes risk loci

#### Type 2 diabetes risk alleles associated with pancreatic beta cell function

**Fig. 3:**
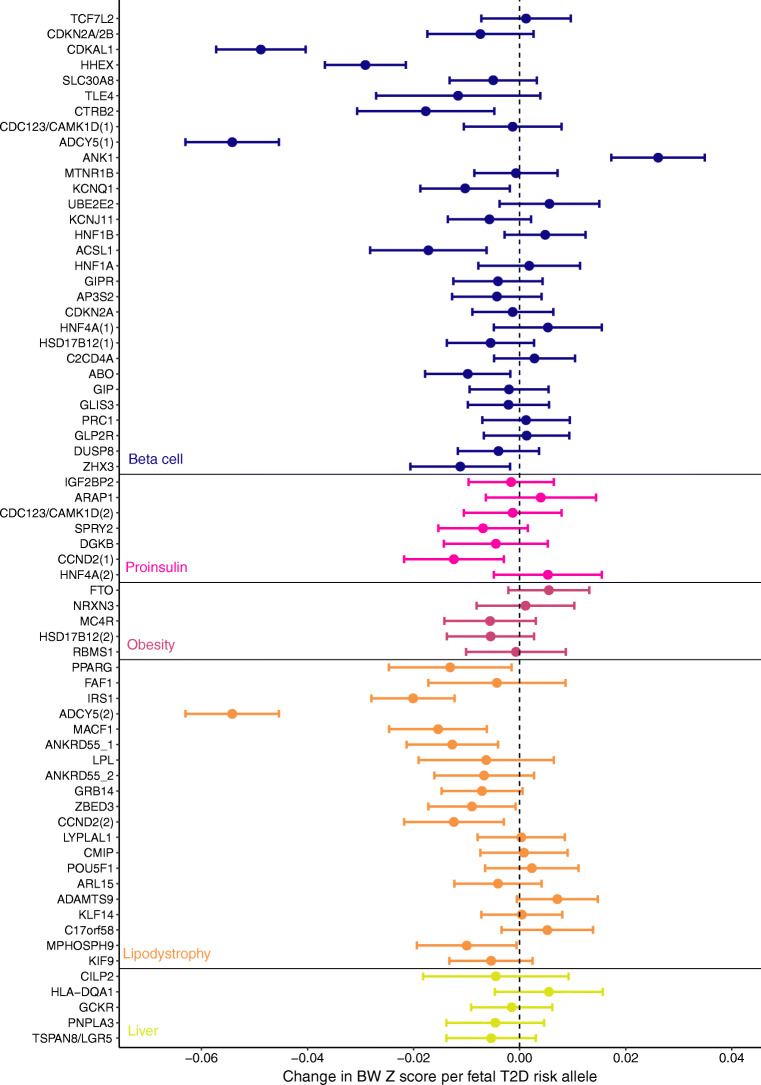
The effect of fetal type 2 diabetes (T2D) risk alleles on birthweight (BW) clustered by their likely underlying biology (beta cell function, proinsulin secretion and insulin resistance secondary to obesity, lipodystrophy-like fat distribution or disrupted liver lipid metabolism) [81]. SNPs within each cluster are ordered from top to bottom by highest to lowest T2D risk (established from a genome-wide association study of participants of European ancestry [61]). SNPs that appear in more than one cluster (*ADCY5*, *CCND2*, *CDC123/CAMK1D*, *HSD17B12*, *HNF4A*) are shown by an accompanying number in parentheses. There are two distinct signals at *ANKRD55* (shown as *ANKRD55_1* and *ANKRD55_2*). The error bars show the 95% CIs for the estimated fetal effect on birthweight in Europeans (independent of any maternal effect [59]), with 1 SD change in birthweight being equivalent to ~450 g. This figure is available as part of a downloadable slideset

The strongest associations between type 2 diabetes risk alleles and lower birthweight are at loci that primarily affect pancreatic beta cell function (e.g. *ADCY5* and *CDKAL1*; Fig. 3). However, not all risk alleles at beta cell loci are associated with lower birthweight. For example, the fetal risk allele at *TCF7L2,* which has a relatively large effect on type 2 diabetes risk, has no effect on birthweight, and the fetal risk allele at the *ANK1* locus is associated with a higher birthweight [59] despite its role in regulating *NKX6-3* [61], a vital transcription factor involved in pancreatic beta cell development [62]. These emerging patterns of association are consistent with the heterogeneous birthweight effects of monogenic causes of diabetes secondary to reduced insulin secretion and suggest that different susceptibility loci exert their effects on beta cell function at different stages in the life course.

#### Type 2 diabetes risk alleles associated with insulin resistance, obesity or liver lipid metabolism

Certain type 2 diabetes risk alleles associated with insulin resistance secondary to a metabolically unfavourable lipodystrophy-like fat distribution (e.g. *IRS1*) are associated with lower birthweight but those implicated in obesity or liver lipid metabolism are not. Consistent with this, recent evidence shows that fetal carriage of variants associated with adult adiposity and a favourable metabolic profile (including higher insulin sensitivity) [63] is associated with higher birthweight [64]. This could mean that a genetic predisposition to lower insulin sensitivity results in a lower birthweight but, in keeping with the monogenic and epidemiological data, the different pathways affecting insulin action are not consistently shared between birthweight and type 2 diabetes risk (Fig. 3).

### Quantifying the relationship between lower birthweight and type 2 diabetes that can be attributed to genetic risk

While there is now clear support for the fetal insulin hypothesis, the question remains as to how much of the association between lower birthweight and type 2 diabetes is explained by the genetic associations. Most variants in the type 2 diabetes risk loci do not appear to be associated with birthweight and the finding that a fetal genetic score for birthweight predominantly influences pathways independent of fetal insulin secretion [65] suggests that a substantial proportion of the fetal genetic variability underlying birthweight does not overlap with underlying susceptibility to type 2 diabetes. However, it remains uncertain how much of the relationship (the covariance) between lower birthweight and type 2 diabetes could be explained by the genetic factors that do overlap. To date, using genome-wide data, shared genetic effects of common variants have been estimated to explain 36% (15–57%) of the negative covariance between birthweight and type 2 diabetes risk [59], although this comes with the important caveat of uncertainty introduced by the likely non-linear relationship between the two phenotypes [57].

### Mendelian randomisation studies exploring the role of the intrauterine environment in determining relationships between lower birthweight and adult cardiometabolic disease

While there is accumulating evidence for the relationship between lower birthweight and type 2 diabetes having a shared genetic aetiology, long-lasting effects of the intrauterine environment on early development are thought to play an important role. Many studies of animal models have shown this to be the case [66] and the most convincing evidence in humans has come from studies of offspring born during periods of famine, showing that they are at a higher risk of disorders of glucose metabolism and type 2 diabetes in adulthood (reviewed in detail in [67]). In addition, monozygotic twins discordant for type 2 diabetes have a lower birthweight [68], a finding which supports an effect of the intrauterine environment on both restricted fetal growth and developmental programming of metabolism.

Genetics can be used to test whether there is a causal relationship between an intrauterine exposure and adult type 2 diabetes by analysing genetic variants specifically associated with the exposure in a technique called Mendelian randomisation [69]. It is akin to a randomised control trial, since genetic variants are randomly assigned at birth and as the genes are specific to the exposure it is not generally subject to confounding from other factors that may mediate the relationship between the exposure and outcome.

**Fig. 4:**
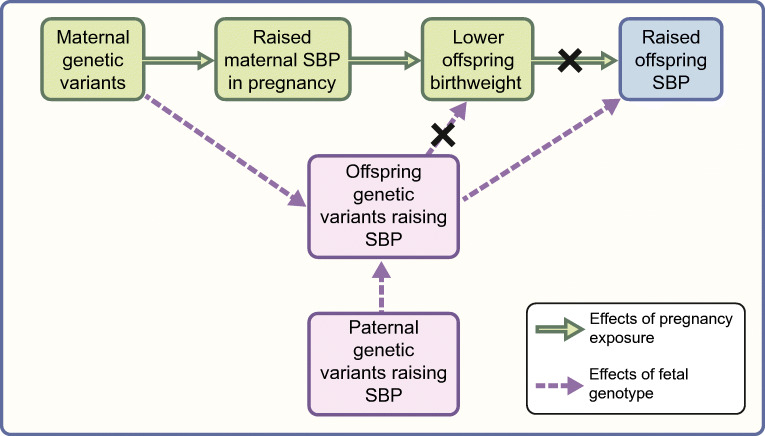
Principles of using Mendelian randomisation to explore the roles of pregnancy exposures and fetal genetics in the relationship between birthweight and risk of adult cardiometabolic disease. The example in this figure shows that the relationship between lower birthweight and higher offspring SBP is mediated by a combination of intrauterine effects on birthweight and fetal genetic susceptibility to higher adult SBP. Figure adapted from Lawlor et al [75] under the terms of the Creative Commons Attribution 4.0 International License (http://creativecommons.org/licenses/by/4.0/), which permits unrestricted use, distribution, and reproduction in any medium. This figure is available as part of a downloadable slideset

There have been attempts to use Mendelian randomisation to show that lower birthweight is causally related to type 2 diabetes [70–72] but the results were difficult to interpret as they did not appropriately differentiate between maternal and fetal effects [73–75]. Methods have been established to account for maternal and fetal effects and test for causal associations between pregnancy exposures and offspring traits [59, 60, 76]. A recent, large study of genotyped parent–offspring pairs (*n*=45,849) showed no evidence for a causal relationship between maternal intrauterine exposures that influence birthweight and offspring quantitative cardiometabolic traits (glucose, lipids, BP, BMI) [76]. A specific example tested by Mendelian randomisation and relevant to the fetal insulin hypothesis is the relationship between maternal systolic BP (SBP) and offspring birthweight and SBP. This showed that while high maternal SBP results in reduced fetal growth, it is not causal for high offspring SBP but instead reflects a shared genetic predisposition to higher SBP (Fig. 4) [59, 76]. This example demonstrates a key underlying premise of the fetal insulin hypothesis: that the fetal genotype can explain observational relationships between lower birthweight and adult traits. However, unlike the fetal insulin hypothesis, the relationship between lower birthweight and higher adult SBP may be explained by a combination of maternal intrauterine effects on birthweight and fetal genetic susceptibility to higher adult SBP.

## Conclusion

In the two decades since the fetal insulin hypothesis was first proposed, advances in genetic research have shed light on what contributes to fetal insulin-mediated growth and its implications for long-term risk of type 2 diabetes. Strong evidence from monogenic studies has been supported by epidemiological observations and discoveries arising from large-scale GWAS of type 2 diabetes and birthweight. Taken as a whole, it is clear that both lower birthweight and type 2 diabetes reflect, in part, a shared genetic predisposition to reduced insulin secretion. However, while impaired insulin action was considered a key part of the original fetal insulin hypothesis, studies of birthweight relating to monogenic lipodystrophies, paternal insulin resistance and the biology underlying shared birthweight and type 2 diabetes risk loci suggest this may be a less important factor in mediating the relationship between lower birthweight and type 2 diabetes risk.

Research investigating the premise of the fetal insulin hypothesis will continue to be important as type 2 diabetes becomes more prevalent globally. As this is predominantly associated with rising levels of obesity, it is possible that the variance in adult type 2 diabetes risk that can be explained by genes which also reduce insulin-mediated fetal growth becomes less important. This is because risk variants associated with high BMI are not strongly represented in birthweight GWAS and mothers with higher BMIs are at risk for diabetes in pregnancy, which leads to higher birthweights. Addressing this and other important challenges, including diversifying research to include non-European populations and exploring non-linear relationships and gene–environment interactions, will provide further insights into the genetics of insulin-mediated fetal growth and its implications for health and disease across the life course.

## Supplementary Information

Slideset of figures (PPTX 476 kb)

## Data Availability

This review did not generate any new data.

## Abbreviations

DOHaD: Developmental Origins of Health and Disease
GWAS: Genome-wide association studies
SBP: Systolic BP
SDS: SD score
