# Supplementary material for: Two decades since the fetal insulin hypothesis: what have we learned from genetics?
Source: Diabetologia. 2021 Feb 11;64(4):717–26. doi: 10.1007/s00125-021-05386-7 (PMC7940336; doi:10.1007/s00125-021-05386-7)
Supplement: Supplementary file 1 — (PPTX 476 kb) [file 125_2021_5386_MOESM1_ESM.pptx]

## Slide 1
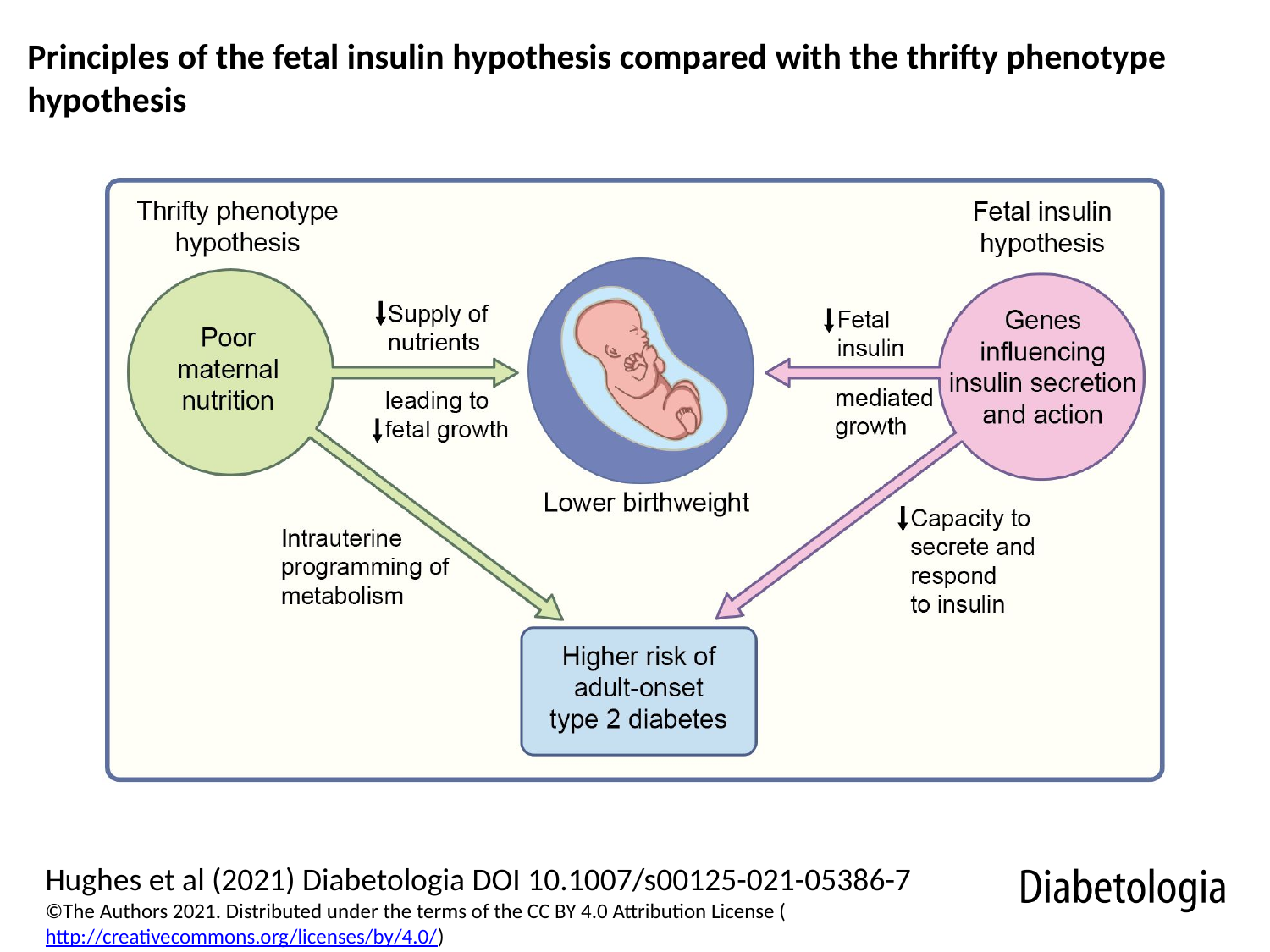

Principles of the fetal insulin hypothesis compared with the thrifty phenotype hypothesis
Hughes et al (2021) Diabetologia DOI 10.1007/s00125-021-05386-7
©The Authors 2021. Distributed under the terms of the CC BY 4.0 Attribution License (http://creativecommons.org/licenses/by/4.0/)

## Slide 2
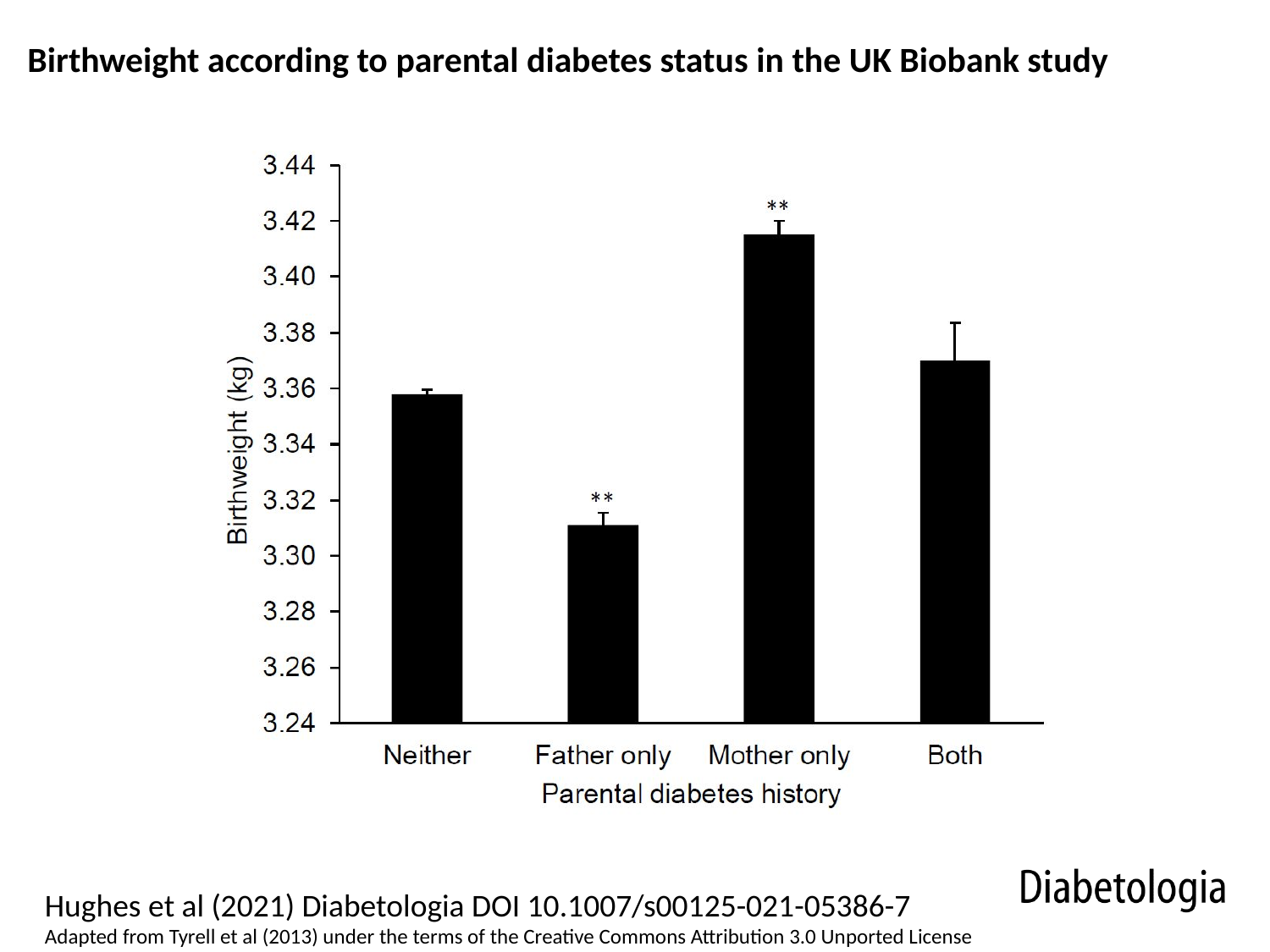

Birthweight according to parental diabetes status in the UK Biobank study
Hughes et al (2021) Diabetologia DOI 10.1007/s00125-021-05386-7
Adapted from Tyrell et al (2013) under the terms of the Creative Commons Attribution 3.0 Unported License

## Slide 3
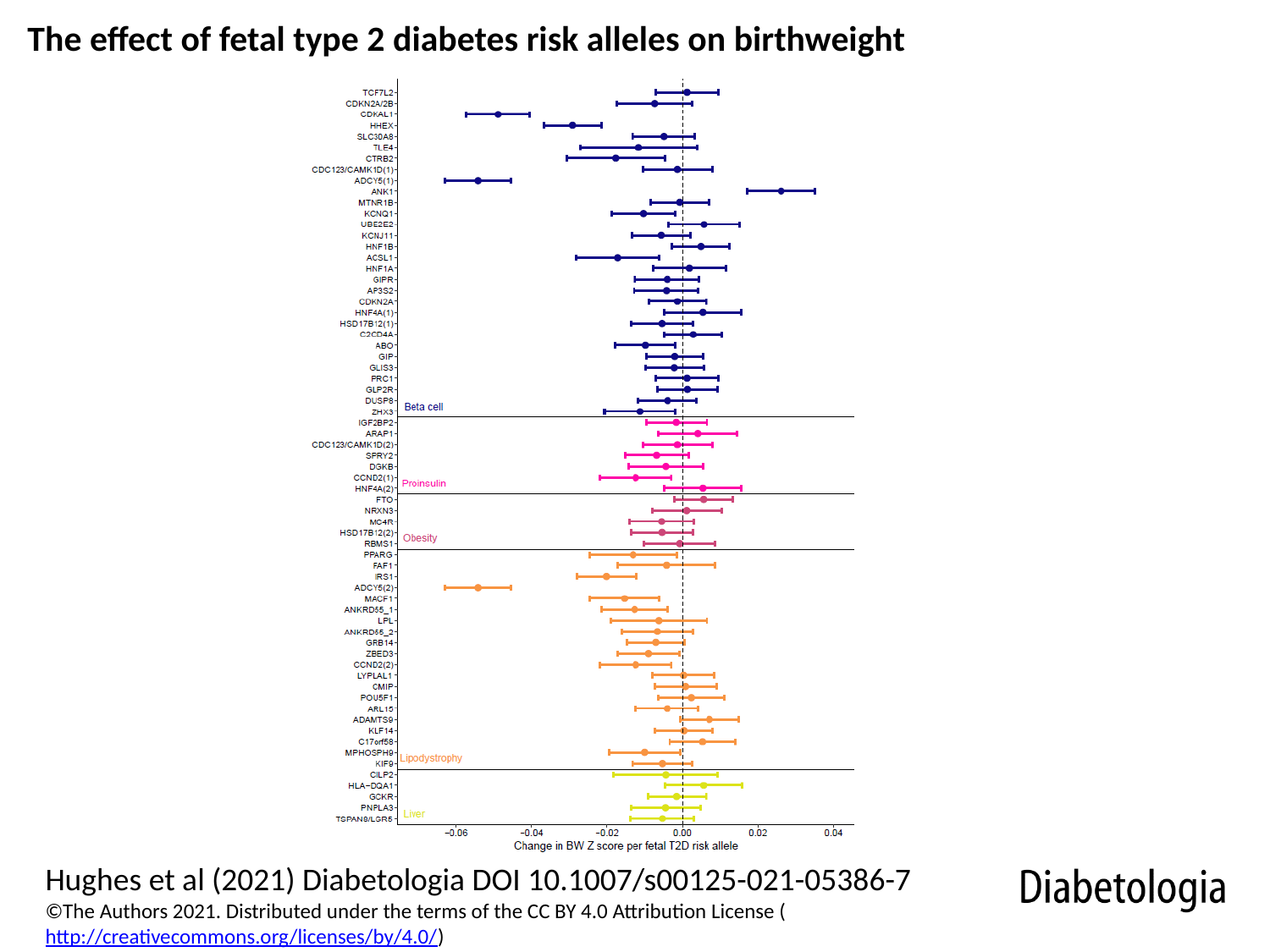

The effect of fetal type 2 diabetes risk alleles on birthweight
Hughes et al (2021) Diabetologia DOI 10.1007/s00125-021-05386-7
©The Authors 2021. Distributed under the terms of the CC BY 4.0 Attribution License (http://creativecommons.org/licenses/by/4.0/)

## Slide 4
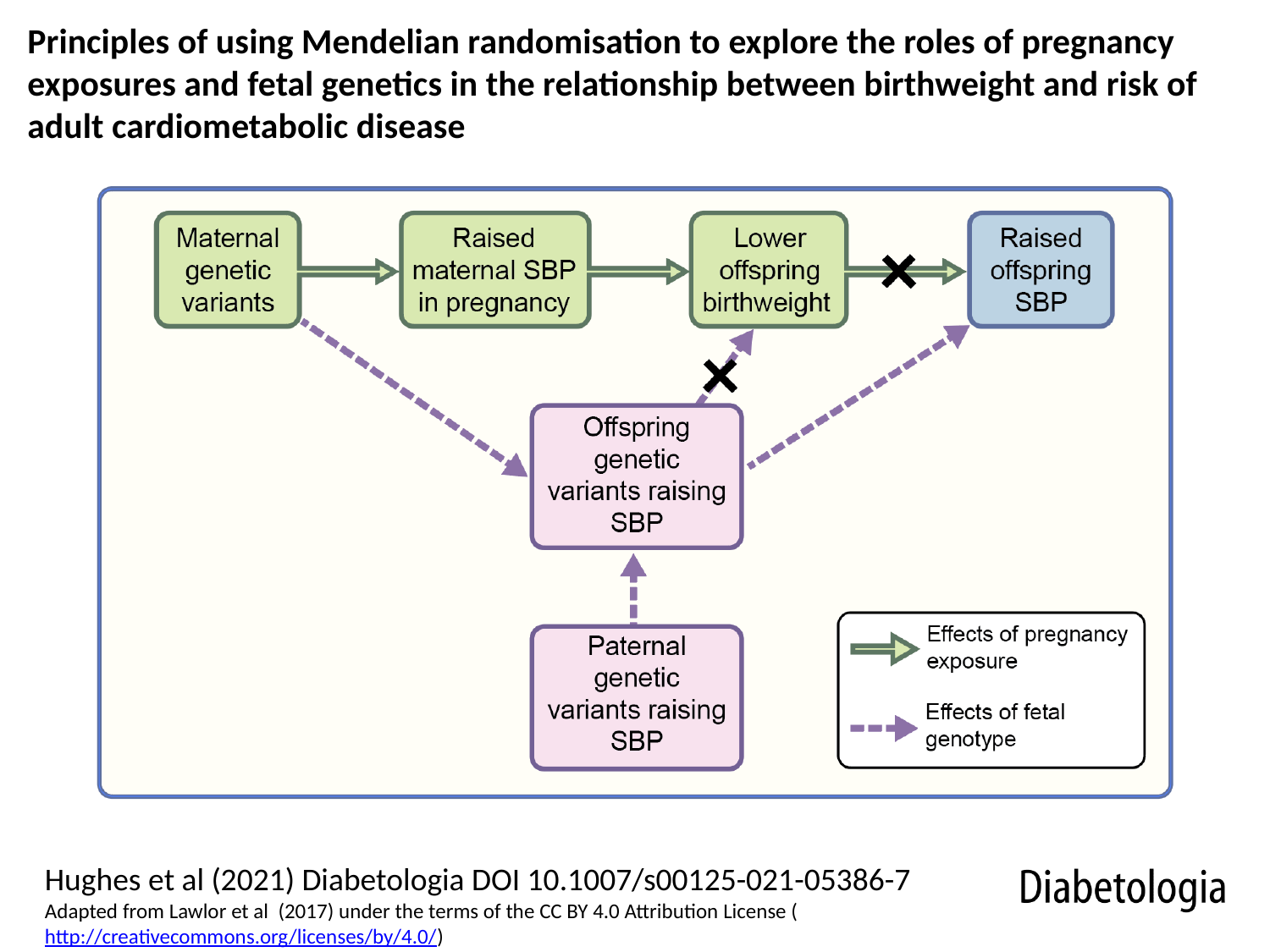

Principles of using Mendelian randomisation to explore the roles of pregnancy exposures and fetal genetics in the relationship between birthweight and risk of adult cardiometabolic disease
Hughes et al (2021) Diabetologia DOI 10.1007/s00125-021-05386-7
Adapted from Lawlor et al (2017) under the terms of the CC BY 4.0 Attribution License (http://creativecommons.org/licenses/by/4.0/)
